# Supplementary material for: Patient and Clinical Factors at Admission Affect the Levels of Neutralizing Antibodies Six Months after Recovering from COVID-19
Source: Viruses. 2022 Jan 2;14(1):80. doi: 10.3390/v14010080 (PMC8779922; doi:10.3390/v14010080)
Supplement: Supplementary file 1 [file viruses-14-00080-s001.zip › viruses-1483482-supplementary.pdf]

Supplementary materials

Patient and clinical factors at admission affect the levels of neutralizing antibodies six months after recovering from COVID-19

Supplementary Table S1. Comparison of demographic and clinical indicators of admission between the NAb^{high} and NAb^{low} groups

Variables	All patients (n=306)	NAb^{high} (n=153)	NAb^{low} (n=153)	P value
**Sex n (%)**				
Male	138 (45.1)	67 (43.8)	71 (46.4)	*0.646*
Female	168 (56.2)	86 (56.2)	82 (53.6)	
**Age, year (IQR)**	62 (53, 68)	64 (54, 69)	61 (53, 67)	*0.133*
**Neutralizing antibody, AU/mL (IQR)**	63.1 (34.7, 108.9)	108.8 (85.0, 161.8)	34.9 (23.1, 48.1)	***<0.0001***
**Severity, n (%)**				
Moderate	174 (56.9)	81 (52.9)	93 (60.8)	*0.060*
Severe	124 (40.5)	65 (42.5)	59 (38.6)	
Critical	8 (2.6)	7 (4.6)	1 (0.7)	
**Symptom, n (%)**				
Fever (temperature ≥ 37.3°C)	191/227 (84.1)	90/110 (81.8)	101/117 (86.3)	*0.353*
Diarrhea	33/226 (14.6)	14/110 (12.7)	19/116 (16.4)	*0.437*

Cough	182/226 (80.5)	91/110 (82.7)	91/116 (78.4)	*0.417*
Fatigue	93/226 (41.2)	49/110 (44.5)	44/116 (37.9)	*0.313*
Chest distress	113/226 (50.0)	59/110 (53.6)	54/116 (46.6)	*0.287*
**Treatment, n (%)**				
Antibiotics	181/293 (61.8)	94/147 (63.9)	85/146 (59.6)	*0.443*
Antiviral	290/294 (98.6)	144/147 (98.0)	146/147 (99.3)	*0.314*
HFNC	12/294 (4.1)	8/147 (5.4)	4/147 (2.7)	*0.238-*
NIV	15/294 (5.1)	10/147 (6.8)	5/147 (3.4)	*0.185-*
IV	1/294 (0.3)	0/147 (0.0)	1/147 (0.7)	*0.316*
CRRT	1/249 (0.4)	0/0 (0.0)	1/116 (0.9)	*0.283*
**Comorbidity, n (%)**				
Hypertension	116/294 (39.5)	60/147 (40.8)	56/147 (38.1)	*0.633*
COLD	12/294 (4.1)	7/147 (4.8)	5/147 (3.5)	*0.556*
CHD	24/294 (8.2)	13/147 (8.8)	11/147 (7.5)	*0.670*
CKD	5/294 (1.7)	3/147 (2.0)	2/147 (1.4)	*0.652*
[a]*Benign tumor*	8/294 (2.7)	5/147 (3.4)	3/147 (2.0)	*0.473*
Other disease	68/293 (23.2)	39/147 (26.5)	29/147 (19.9)	*0.176*
**Physiological parameter, (IQR)**				
Heart rate, times per min	89 (80, 102), n=292	89 (80, 102), n=147	90 (80, 102), n=145	*0.780*
Systolic pressure	131 (120, 143), n=292	128 (120, 141), n=147	132 (121, 144), n=145	*0.174*
Diastolic pressure	80 (73, 89), n=292	80 (72, 88), n=147	81 (75, 90), n=145	*0.188*
RR, breaths per min	20 (19, 21), n=292	20 (19, 22), n=147	20 (20, 21), n=145	*0.494*
**Biological parameters, (IQR)**				
WBC, $\times 10^9$/L	5.4 (4.3, 7.2), n=224	5.6 (4.3, 7.3), n=110	5.2 (4.3, 7.1), n=114	*0.463*
Neutrophil, $\times 10^9$/L	3.8 (2.6, 5.4), n=224	3.8 (2.7, 5.6), n=110	3.7 (2.5, 5.2), n=114	*0.422*
Lymphocyte, $\times 10^9$/L	1.03 (0.73, 1.44), n=225	1.04 (0.74, 1.52), n=110	1.02 (0.73, 1.37), n=115	*0.751*

Hemoglobin, g/L	126 (115, 137), n=222	126 (117, 135), n=109	126 (115, 140), n=113	*0.285*
Platelet, ×10⁹/L	217 (160, 289), n-222	222 (162, 294), n=109	207 (159, 272), n=113	*0.445*
Albumin, g/L	34.2 (30.9, 38.0), n=223	34.2 (30.7, 37.5), n=108	34.3 (31.0, 38.1), n=115	*0.672*
LDH, U/L	274 (214, 363), n=290	285 (219, 400), n=145	261 (206, 342), n=145	*0.067*
$NaHCO_3$, mmol/L	23.9 (21.9, 25.6), n=222	23.9 (22.0, 25.6), n=108	23.9 (21.7, 26.0), n=114	*0.885*
Creatinine, μmol/L	71.0 (59.0, 84.0), n=223	74.0 (60.3, 87.0), n=109	68.5 (58.0, 81.0), n=114	*0.143*
Creatine kinase, U/L	79.0 (43.0, 121.0), n=75	83.0 (43.0, 133.5), n=33	76.0 (42.3, 123.3), n=42	*0.890*
hs-cTnI, pg/mL	4.4 (2.0, 9.6), n=230	4.5 (2.1, 10.3), n=116	4.4 (1.9, 9.5), n=114	*0.434*
IL-2R, U/mL	714.0 (537.0, 1092.0), n=67	747.0 (556.0, 1139.), n=35	712.5 (529.5, 896.5), n=32	*0.514*
IL-6, pg/mL	21.9 (4.1, 46.8), n=68	22.2 (4.3, 53.4), n=35	15.7 (4.0, 42.4), n=33	*0.393*
IL-8, pg/mL	14.8 (8.8, 26.0), n=67	15.0 (8.7, 29.7), n=35	14.6 (9.1, 25.0), n=32	*0.920*
IL-10, pg/mL	5.3 (5.0, 9.3), n=67	6.2 (5.0, 11.4), n=35	5.1 (5.0, 8.8), n=32	*0.338*
NT-proBNP, pg/mL	164.0 (66.3, 422.8), n=144	165.5 (67.8, 425.8), n=85	162.5 (63.8, 413.0), n=76	*0.764*
D-Dimer, μg/mL	0.96 (0.48, 1.75), n=201	1.06 (0.48, 1.76), n=99	0.88 (0.44, 1.75), n=102	*0.391*

[a]Benign tumor, benign tumors or cancers that had been eradicated before admission (including thyroid cancer or early-stage prostate cancer and so on). Data is shown as the median (IQR), n (%), or n/N (%), where N is the total number with the available data. NAb, neutralizing antibody; IQR, interquartile range; HFNC, high-flow nasal cannula oxygen therapy; NIV, non-invasive mechanical ventilation; IV, invasive mechanical ventilation; CRRT, continuous renal replacement therapy; COLD, chronic obstructive lung disease; CHD, coronary heart disease; CKD, chronic kidney disease; RR, respiratory rate; Hs-cTnI, hypersensitive cardiac troponin I; WBC, white blood cell count; NT-proBNP, N terminal pro B type natriuretic peptide; LDH, lactate dehydrogenase.

Supplementary Table S2. Comparison of demographic and clinical indicators of admission between the NAb^{higher} and NAb^{lower} groups

Variables	All patients (n=152)	NAb^{higher} (n=76)	NAb^{lower} (n=76)	P value
**Sex n (%)**				
Male	63 (41.4)	27 (35.5)	36 (47.4)	*0.138*
Female	89 (58.6)	49 (64.5)	40 (52.6)	
**Age, year (IQR)**	63 (53, 68)	65 (55, 70)	62 (52, 67)	*0.037*
**Neutralizing antibody, AU/mL (IQR)**	62.9 (23.0, 155.9)	155.3 (116.6, 200.7)	23.2 (16.2, 30.1)	***<0.0001***
**Severity, n (%)**				
Moderate	90 (59.2)	39 (51.3)	51 (67.1)	*0.101*
Severe	61 (40.1)	36 (47.4)	25 (32.9)	
Critical	1 (0.7)	1 (1.3)	0 (0)	
**Symptom, n (%)**				
Fever (temperature ≥ 37.3°C)	84/106 (79.2)	39/51 (76.5)	45/55 (81.8)	*0.498*
Diarrhea	16/105 (15.2)	7/51 (13.7)	9/54 (16.7)	*0.675*
Cough	82/105 (78.1)	40/51 (78.4)	42/54 (77.8)	*0.935*
Fatigue	43/105 (41.0)	23/51 (45.1)	20/54 (37.0)	*0.401*
Chest distress	50/105 (47.6)	25/51 (49.0)	25/54 (46.3)	*0.780*
**Treatment, n (%)**				
Antibiotics	84/145 (57.9)	43/72 (59.7)	41/73 (56.2)	*0.664*
Antiviral	143/146 (97.9)	69/72 (95.8)	74/74 (100)	*0.076*
IVIG	34/146(23.3)	21/72 (29.2)	13/74 (17.6)	*0.097*
HFNC	7/146 (4.8)	4/72 (5.6)	3/74 (4.1)	*0.671*
NIV	8/146 (5.5)	4/72 (5.6)	4/74 (5.4)	*0.968*
**Comorbidity, n (%)**				

Hypertension	51/146 (34.9)	27/72 (37.5)	24/74 (32.4)	*0.521*
COLD	6/146 (4.1)	4/72 (5.6)	2/72 (2.7)	*0.385*
CHD	16/146 (11.0)	10/72 (13.9)	6/74 (8.1)	*0.264*
CKD	2/146 (1.4)	1/72 (1.4)	1/74 (1.4)	*0.984*
[a]*Benign tumor*	3/146 (2.1)	3/72 (4.2)	0/74 (0.0)	*0.076*
Other disease	32/145 (22.1)	17/72 (23.6)	15/73 (20.5)	*0.657*
**Physiological parameter, (IQR)**				
Heart rate, times per min	89 (80, 100), n=145	89 (80, 102), n=72	89 (80, 99), n=73	*0.862*
Systolic pressure	131 (121, 145), n=145	130 (120, 141), n=72	132 (122, 147), n=73	*0.255*
Diastolic pressure	80 (74, 89), n=145	79 (71, 88), n=72	81 (76, 90), n=73	*0.182*
RR, breaths per min	20 (20, 22), n=145	20 (20, 22), n=72	20 (19, 21), n=73	*0.204*
**Biological parameters, (IQR)**				
WBC, ×10^9/L	5.0 (4.3, 7.1), n=104	5.0 (4.1, 7.3), n=51	5.0 (4.4, 6.9), n=53	*0.917*
Neutrophil, ×10^9/L	3.5 (2.5, 5.3), n=105	3.5 (2.5, 5.6), n=5U1	3.6 (2.5, 4.7), n=54	*0.865*
Lymphocyte, ×10^9/L	1.12 (0.80, 1.50), n=105	1.14 (0.80, 1.55), n=51	1.07 (0.76, 1.40), n=54	*0.783*
Hemoglobin, g/L	127 (116, 137), n=104	126 (118, 134), n=51	130 (115, 141), n=53	*0.323*
Platelet, ×10^9/L	212 (164, 281), n-104	224 (167, 289), n=51	206 (164, 266), n=53	*0.518*
Albumin, g/L	34.3 (30.6, 38.2), n=105	34.0 (29.6, 36.9), n=51	35.2 (31.3, 38.6), n=54	*0.121*
LDH, U/L	276.0 (214, 360.5), n=145	295 (220, 404), n=72	255 (199, 338.5), n=73	*0.114*
$NaHCO_3$, mmol/L	24.4 (21.9, 26.1), n=103	24.1 (22.1, 25.9), n=50	24.7 (21.8, 26.4), n=53	*0.566*
Urea, mmol/L	4.4 (3.45, 5.9), n=104	4.7 (3.5, 6.5), n=51	3.9 (3.4, 5.6), n=53	*0.145*
Creatinine, µmol/L	67.0 (58.3, 83.3), n=104	67.0 (56.0, 86.0), n=51	67.0 (59.0, 81.0), n=53	*0.812*
Creatine kinase, U/L	82.0 (52.0, 149.0), n=35	80.0 (42.3, 147.0), n=16	82.0 (53.0, 149.0), n=19	*0.679*
SF, µg/L	537.2 (297.5, 1228.8), n=39	744.1 (387.9, 1360.8), n=19	449.6 (184.8, 744.0), n=20	*0.106*
hs-cTnI, pg/mL	4.5 (2.4, 10.0), n=113	4.6 (2.3, 10.3), n=56	4.0 (2.4, 8.4), n=57	*0.386*
IL-2R, U/mL	713.0 (527.5, 941.5), n=33	701.5 (535.0, 1168.3), n=16	713.0 (519.5, 802.0), n=17	*0.614*

IL-6, pg/mL	16.6 (4.0, 35.6), n=34	22.6 (4.0, 68.1), n=16	10.7 (3.7, 24.9), n=18	*0.190*
IL-8, pg/mL	15.0 (11.8, 24.1), n=33	19.3 (11.5, 32.8), n=16	14.4 (10.1, 20.7), n=17	*0.288*
IL-10, pg/mL	5.0 (5.0, 9.2), n=33	6.5 (5.0, 10.2), n=16	5.0 (5.0, 7.4), n=17	*0.138*
NT-proBNP, pg/mL	191.0 (73.5, 478.5), n=69	214.0 (81.5, 551.0), n=33	176.0 (66.5, 466.0), n=36	*0.417*
D-Dimer, μg/mL	0.95 (0.43, 2.04), n=97	1.18 (0.48, 2.21), n=47	0.66 (0.42, 1.89), n=50	*0.231*

[a]Benign tumor, benign tumors or cancers that had been eradicated before admission (including thyroid cancer or early-stage prostate cancer and so on). Data is shown as the median (IQR), n (%), or n/N (%), where N is the total number with the available data. NAb, neutralizing antibody; IQR, interquartile range; IVIG, intravenous immunoglobin; HFNC, high-flow nasal cannula oxygen therapy; NIV, non-invasive mechanical ventilation; COLD, chronic obstructive lung disease; CHD, coronary heart disease; CKD, chronic kidney disease; RR, respiratory rate; SF, serum ferritin; Hs-cTnI, hypersensitive cardiac troponin I; WBC, white blood cell count; NT-proBNP, N terminal pro B type natriuretic peptide; LDH, lactate dehydrogenase.

Supplementary Table S3. Cutoff values for the dichotomous categories in the ROC analysis of the continuous variables before GMM model

Variables	Cutoff of dichotomous categories
SpO_2, %	96.5
LDH, U/L	322.5
Urea, mmol/L	4.65
CRP, mg/L	57.35
PCT, ng/mL	0.055
Fibrinogen, g/L	5.765

ROC, receiver operating characteristic; GMM, Gaussian mixture model; SpO_2, pulse oxygen saturation; PCT, procalcitonin; CRP, C-reactive protein; LDH, lactate dehydrogenase.

Supplementary Figure S1.

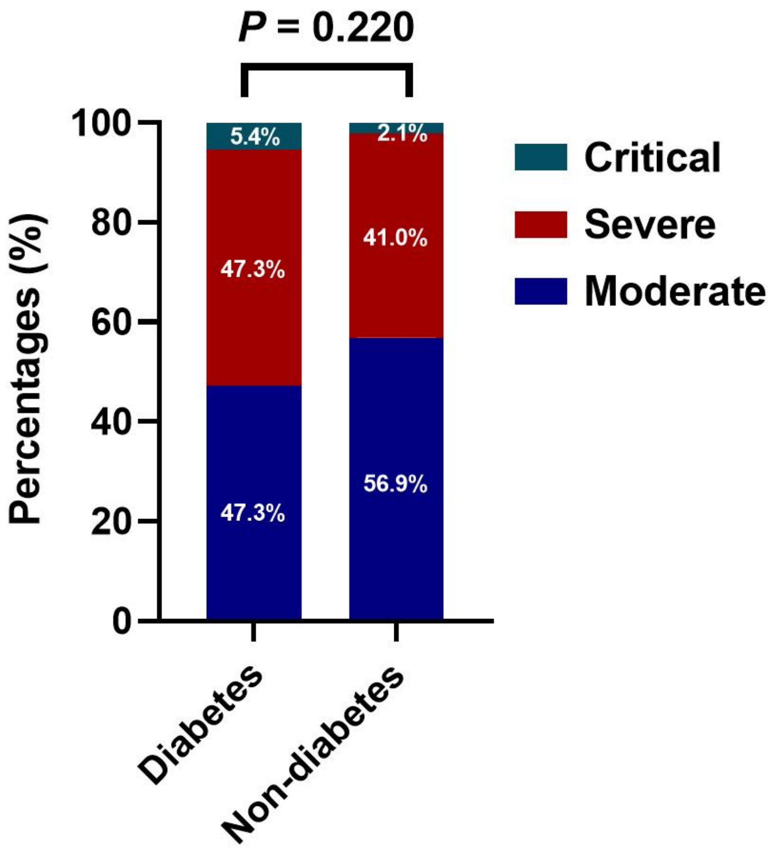

Supplementary Figure S1. Distribution of disease severity in diabetic and non-diabetic patients among 306 patients recovered from COVID-19. No significant difference in disease severity was found between patients with diabetes and non-diabetes. In patients with diabetes, there were 26 cases in moderate (47.3%), 26 in severe (47.3%) and 3 (5.4%) in critical severity whereas in non-diabetes patients, there were 136 cases in moderate (56.9%), 98 in severe (41.0%) and 5 (2.1%) in critical severity, p = 0.220. NAb, neutralizing antibody.

Supplementary Figure S2.

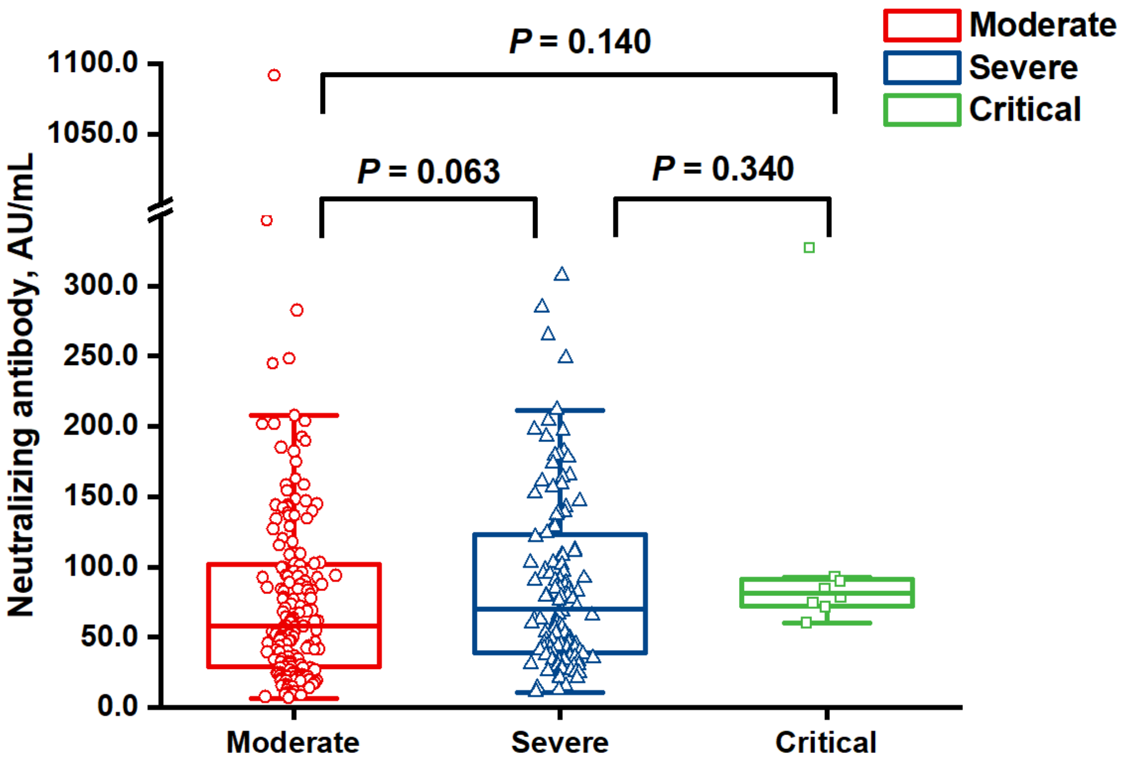

Supplementary Figure S2. NAb levels in patients with different disease severity. NAb levels did not exhibit significant differences overall in patients of different disease severity. NAb levels in moderate patients (median (IQR): 57.9 (28.9, 102.3) AU/mL), n=174; NAb levels in severe patients (median (IQR): 69.7 (39.0, 123.5) AU/mL), n=124; NAb levels in critical patients (median (IQR): 81.2 (71.8, 91.9) AU/mL), n=8. NAb, neutralizing antibody.
